# Supplementary material for: DMRforPairs: identifying Differentially Methylated Regions between unique samples using array based methylation profiles
Source: BMC Bioinformatics. 2014 May 15;15:141. doi: 10.1186/1471-2105-15-141 (PMC4046028; doi:10.1186/1471-2105-15-141)
Supplement: Additional file 2 — DMRforPairs output for the comparison of A431-MCF7 and NA17018-NA17105. Please start from the HTML files in each folder. Available via the BMC Bioinformatics website. [file 1471-2105-15-141-S2.zip › 1394847754114233_MOESM2_ESM/A431_MCF7/figures/30070.pdf]

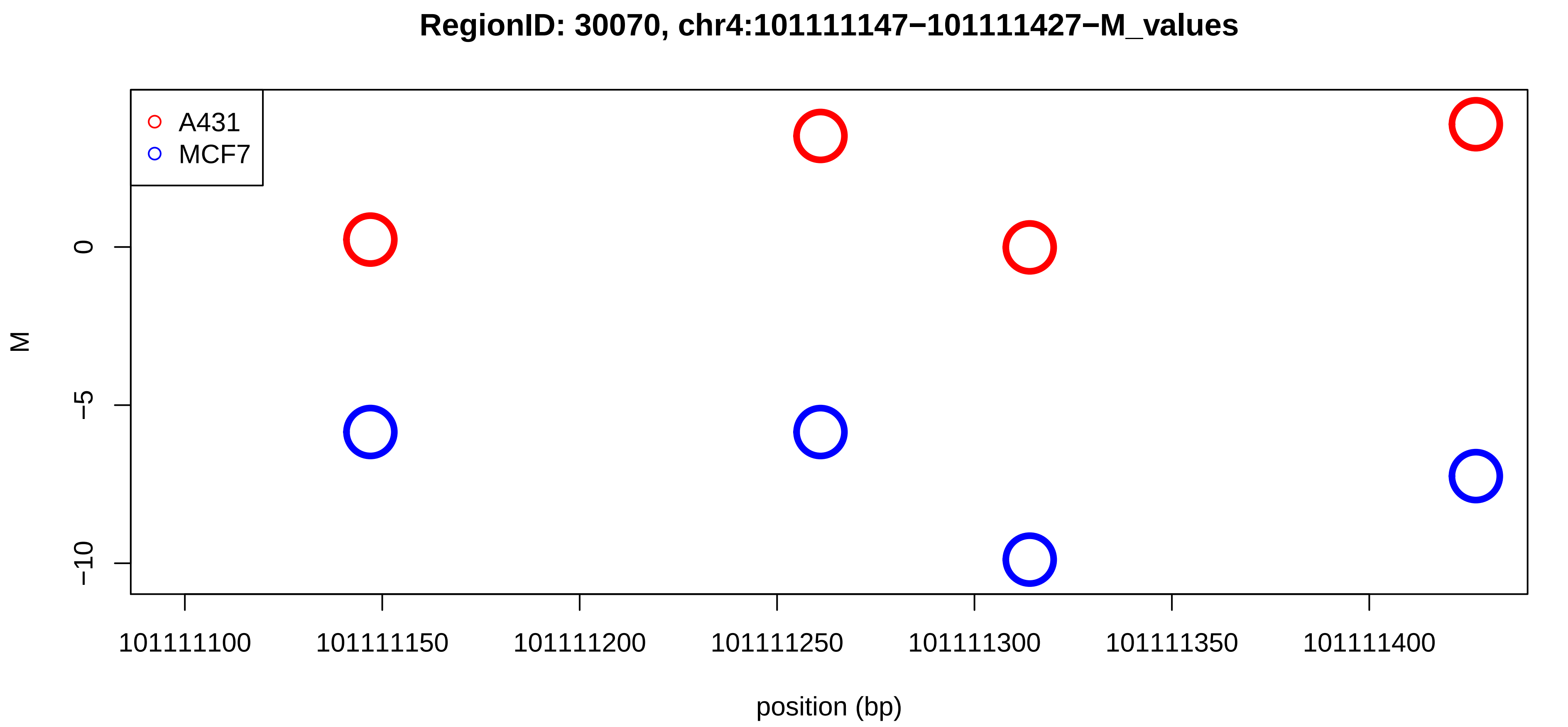
RegionID: 30070, chr4:101111147−101111427−M_values
A431
MCF7
M
0
−5
−10
101111100
101111150
101111200
101111250
101111300
101111350
101111400
position (bp)

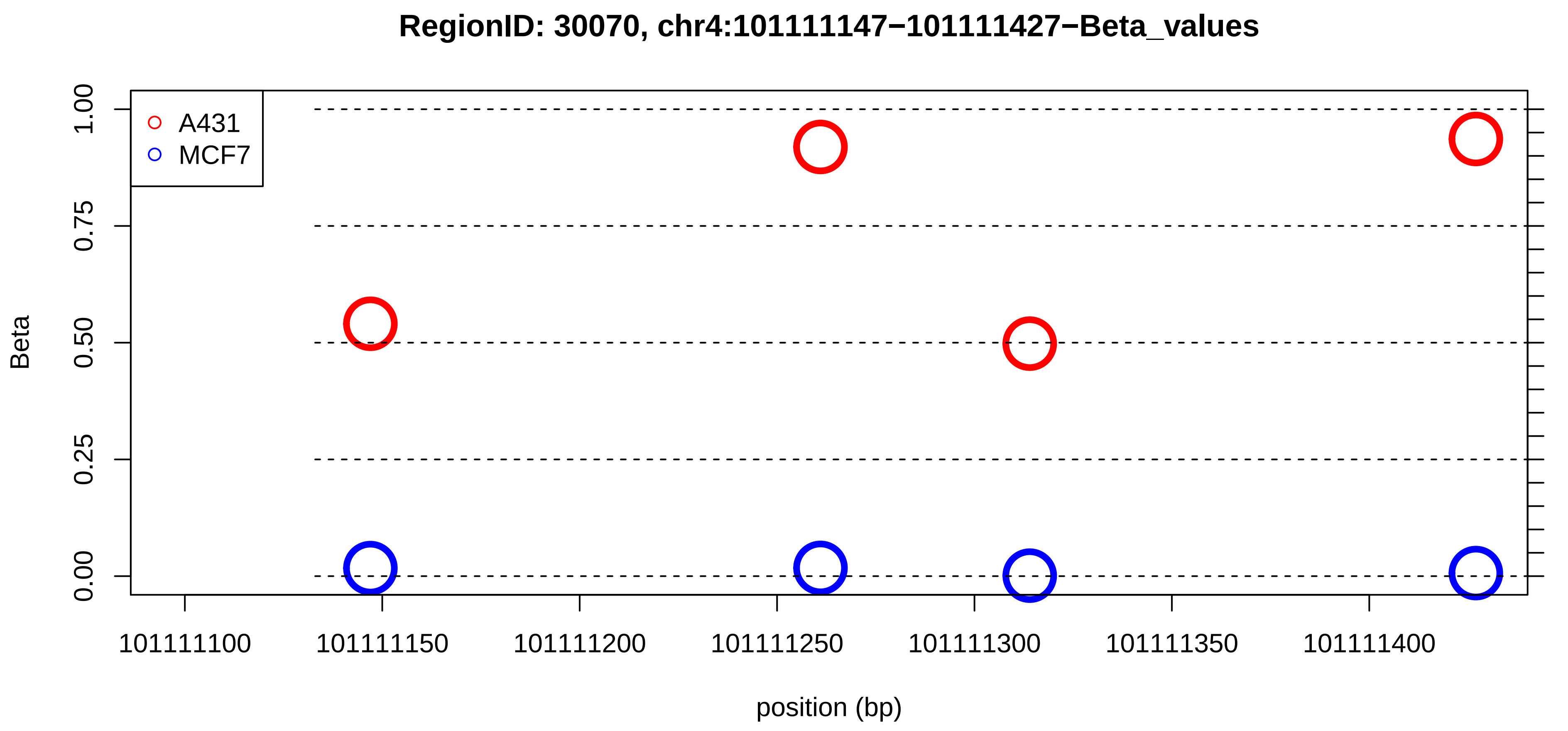
RegionID: 30070, chr4:101111147−101111427−Beta_values
A431
MCF7
Beta
1.00
0.75
0.50
0.25
0.00
101111100
101111150
101111200
101111250
101111300
101111350
101111400
position (bp)
